# Investigation of ceramic MFC stacks for urine energy extraction

**DOI:** 10.1016/j.bioelechem.2018.03.010

**Published:** 2018-10

**Authors:** Asimina Tremouli, John Greenman, Ioannis Ieropoulos

**Affiliations:** a Bristol BioEnergy Centre, BRL, University of the West of England, T-Block, Frenchay Campus, Bristol BS16 1QY, UK; b School of Chemical Engineering, National Technical University of Athens, Heroon Polytechniou 9, 15780 Zografou, Athens, Greece

**Keywords:** MFC, Stack, Voltage reversal, Ceramic, Urine

## Abstract

Two ceramic stacks, terracotta (t-stack) and mullite (m-stack), were developed to produce energy when fed with neat undiluted urine. Each stack consisted of twelve identical microbial fuel cells (MFCs) which were arranged in cascades and tested under different electrical configurations. Despite voltage reversal, the m-stack produced a maximum power of 800 μW whereas the t-stack produced a maximum of 520 μW after 62.6 h of operation. Moreover, during the operation, both systems were subject to blockage possibly due to struvite. To the Authors' best knowledge, this is the first time that such a phenomenon in ceramic MFC membranes is shown to be the direct result of sub-optimal performance, which confirms the hypothesis that ceramic membranes can continue operating long-term, if the MFCs produce maximum power (high rate of e^−^ transfer). Furthermore, it is shown that once the ceramic membrane is blocked, it may prove difficult to recover *in-situ*.

## Highlights

• Mullite ceramic outperformed terracotta.
• Deficiency in performance reversed polarity for connected cells.
• Ceramic membranes blocked due to suboptimal charge transfer rates
• Blockage in these ceramics was irreversible in situ.

## 1 Introduction

Microbial fuel cells (MFCs) are systems that convert biomass directly into electricity through the metabolic activity of microorganisms [1,2]. In recent years, the interest for this technology has rapidly increased, since MFCs offer the advantage of simultaneous treatment of wastewater and energy generation in the form of electricity [3,4]. Several MFC designs have been reported with optimised parameters for power production and wastewater treatment, when fed with different types of feedstock [2,5,6].

In order to better exploit the advantages offered by MFCs, system configurations must be operationally and economically sustainable. Although several bioreactor designs have been investigated, under different operating conditions and both expensive and cheap materials have been tested with various substrates, the MFC technology has still not been commercialised [2,5]. The main obstacles that this technology has to overcome are the low energy generated when compared to more conventional mature technologies such as chemical fuel cells, batteries, photovoltaics (although the per-unit power output can be comparable to single PV cells that collectively make up a PV panel) as well as the high cost of some of the materials used [7,8].

Stacking MFCs could enhance power production and wastewater treatment efficiency. However, there may be challenges when connecting multiple units together as this can be done in a number of different ways (series, parallel and series/parallel combined). Multiple MFC connection could result in losses via the conductive fluidic connections of interconnected units and it can affect the performance depending on whether or not feeding is in series from a common tank or individually fed from the source [[9], [10], [11], [12], [13]]. Moreover, the material selection for the optimisation of the MFC performance is of utmost importance. In order for MFCs to be widely deployed, research towards the effective use of inexpensive and sustainable materials must be carried out. Recently, researchers have started using ceramics with encouraging results, suggesting that these inexpensive materials may be the solution towards MFC implementation. There have even been reports of MFCs made from paper and other biodegradable materials, which in a way opens up new opportunities for low-cost, fixed-term MFC deployment [14,15]. Studies have demonstrated that ceramics can provide stability, improve power and treatment efficiency, create a better environment for the electro-active bacteria to achieve resource recovery and even kill pathogens [6,[16], [17], [18]].

Urine is an abundant waste product and it has already been reported as an excellent fuel for generating electricity in MFCs [19]. The daily production of human urine is estimated to be in the range of 17.4 billion L, based on a world population of 6.97 billion and considering that an adult produces an average of 2.5 L in a day [19,20].

The main objective of this study was therefore to investigate the performance of MFCs when operated under adverse conditions in order to evaluate the limits of the ceramic separators. Two ceramic stacks were constructed and operated under sub-optimal feed/flow conditions, whilst efforts to optimise power production were attempted. MFCs within the stack were fed from a common tank with a dripping mechanism. Neat, urine was used as a feedstock whilst ceramic (terracotta and mullite) was used as (i) the structural material and (ii) the separator for ion exchange. To the Authors' best knowledge, this is the first time that membrane blockage is shown to be the direct result of MFC under-performance. It is also shown that once the ceramic membrane is blocked it may be difficult to recover in-situ.

## 2 Materials and methods

### 2.1 MFC design

**Fig. 1:**
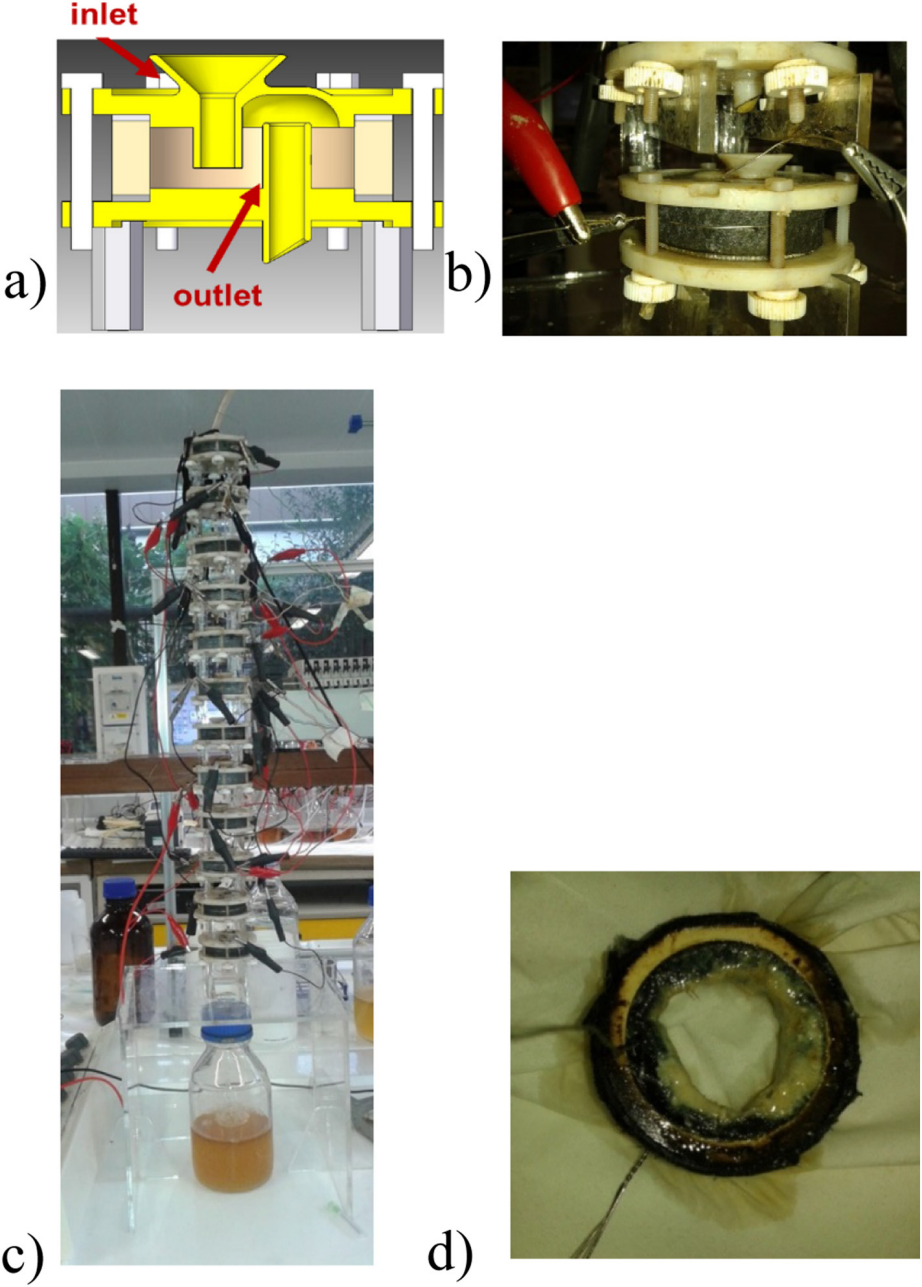
(a) Cross section diagram (b) photo of the MFC (c) photo of the cascade stack (d) photo of the anode chamber after 756.1 h of cell operation.

Each MFC unit consisted of a single cylindrical ceramic chamber, whose internal volume was used as the anode chamber, and the external surface was used as the open-to-air cathode. Two circular 3D printed lids made from acrylo-nitrile butadiene styrene (ABS) material sealed the top and the bottom of the chamber. The inlet was at the top, with the feedstock flowing towards the bottom of the anodic chamber, for better fluid percolation. The effluent went out from the top of the anodic chamber, with the use of a constant-level outlet tube. A schematic cross section and a photo of the unit are shown in Fig. 1a and b.

Untreated carbon fibre veil material (PRF Composites, Dorset, UK) with a density of 20 g/cm^2^ and a total surface area of 64.8 cm^2^ was folded and used as the anode electrode. The cathode electrode (13.75 cm^2^) was prepared by coating activated carbon (AC) (G Baldwin & Co) paste on polytetrafluoroethylene (PTFE) (60 wt% Sigma-Aldrich) treated carbon cloth and was tightly wrapped around the outside wall of the ceramic, with the AC side facing the ceramic material. The paste was prepared by mixing AC powder, PTFE solution and distilled water (80 g/140 mL), as previously described [21]. Stainless steel wire (0.5 mm, Scientific Wire Company) was threaded through the electrodes, which were connected with stainless steel crocodile clips acting as current collectors.

### 2.2 MFC stacking

Two stacks were fabricated and each one consisted of twelve identical MFCs as previously described (Fig. 1). The units in the stack were arranged in cascades such that the effluent from the upstream MFC flowed as the influent into the downstream MFC. MFCs within the cascades were connected electrically together and a dripping mechanism (to introduce an air-gap) was used for fluidic isolation and thus electrical insulation between MFCs. Mullite (Anderman Ceramics Ltd, Hartlebury Trading Estate, Hartlebury, UK) was used for the first stack (m-stack) and terracotta (Weston Mill Pottery, Sutton on Trend, UK) for the second stack (t-stack). The height of the terracotta cylinders was 11 mm with 3 mm wall thickness, whilst mullite cylinders had 11.5 mm height and 5 mm wall thickness. All cylinders had an external diameter of 40 mm and an open porosity of approximately 27%.

### 2.3 MFC enrichment and operation

The two stacks were operated under identical conditions in order to compare the performance of the two ceramic materials. The enrichment and adaptation of the electrochemically active bacteria in the MFCs were performed in batch mode, under a fixed external load of 2 kΩ for each cell. During inoculation, 50% of activated sewage sludge supplied from the Wessex Water Scientific Laboratory (Saltford, UK) and 50% of fresh urine was used as the feedstock. Urine was collected from healthy individuals aged between 18 and 70 years old, with no known medical conditions. Following the enrichment of cells, the operation was shifted to continuous mode, using only fresh urine as the feed. The first unit placed at the top of each stack was being fed directly from the inlet reservoir using a peristaltic pump (205 U, Watson Marlow, Falmouth, UK). The 2 kΩ external resistive loads were removed and a 1 kΩ load was connected to each stack. Due to practical limitations such as urine availability, the MFCs were operated at a sub-optimally slow flow rate 7.51 ml/h (HRT 0.8 h). The anodic liquid volume was 4 ml during batch operation. This volume gradually decreased to approximately 1 ml, as a result of struvite precipitation, probably due to the slow flow rate.

In an attempt to improve performance and recover power to at least the levels produced at the start of the experiment, a series of tests and parameter changes were performed such as: hydration of the cathode electrodes with deionized water, changing the electrical configuration, changing the flow rate, as well as replacing all the cathode electrodes with new identical ones. Moreover, the cells were disassembled and the struvite, which had accumulated on the anode electrodes, was removed. The final step was to examine if the ceramic material was blocked and for this the anode and cathode electrodes were replaced with new identical ones, but keeping the same ceramic chassis. All experiments were performed at room temperature (22 ± 2 °C).

### 2.4 Data collection

Electrode output for each MFC was individually recorded in volts (V) versus time using an Agilent data logger (KEYSIGHT, 34972A LXI data acquisition/Switch) unit. The current and power produced from the MFCs were calculated using Ohm's law *I* = *V* / *R*, where *V* is the measured voltage in volts (*V*) and *R* is the known value of the external load resistor in ohms (Ω). Power (*P*) in watts (W) was calculated by multiplying voltage with current; *P* = *I* × *V*. Power densities were calculated by dividing output with the anodic liquid volume (4 ml for individual MFCs).

### 2.5 Polarisation experiments

The polarisation experiments were performed using a decade variable resistor box (Centrad Boite A Decades De Resistances DR07). Data were produced by sweeping resistor values from 1 MΩ to 0 Ω. The time interval between resistance changes was 3 min and two different electrical configurations were assessed. During the first electrical connection, the top four (1–4), the middle four (5–8) and the bottom four MFCs (9–12), where connected in parallel, and the three parallel groups were connected in series (3s4p). During the second electrical configuration MFCs 1, 2, 11, 12; 3, 4, 9, 10 and 5, 6, 7, 8, were connected in parallel, and the three respective parallel groups were then connected in series. MFCs were named based on their position in the cascade i.e. from MFC1 (the first to receive feedstock) up to MFC12 (the last to receive feedstock).

## 3 Results and discussion

### 3.1 MFCs start-up

In order to inoculate the anodic electrodes with electroactive bacteria, the MFC units were initially operated in batch mode. The beginning of each cycle was marked by the feedstock replacement with 50% of fresh activated sewage sludge and 50% of fresh urine whilst the end of a cycle was marked by the MFC voltage dropping to approximately zero. Following, a relatively reproducible cell performance for the last cycles, the twelve identical MFCs were arranged in cascades and fed in continuous mode (7.51 ml/h). During the batch and continuous mode operations, each MFC unit operated under a fixed external load of 2 kΩ.

**Fig. 2:**
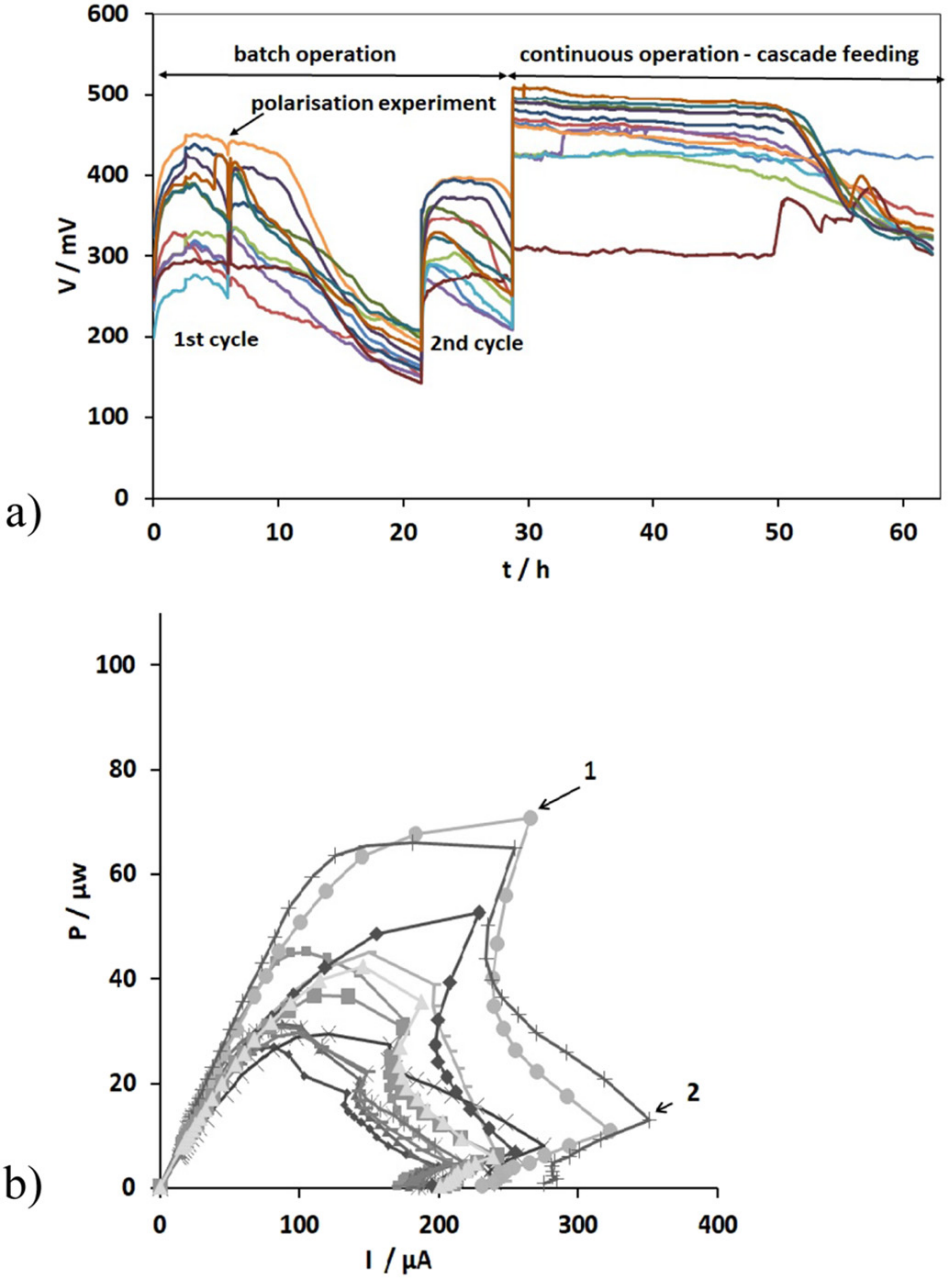
(a) Voltage behaviour of the m-units versus time during the start-up phase (m1: , m2: , m3: , m4: , m5: , m6:, m7:, m8: , m9: , m10:, m11:, m12: ) (b) power curves of the m-units for the first reproducible batch cycle (recorded at t = 5.9 h) (m1: , m2: , m3: , m4: , m5: , m6: , m7: , m8: , m9: , m10: , m11: , m12: ).

Fig. 2a shows the changes in the monitored cell voltage during the start-up stage for the last two reproducible batch cycles and the cascade feeding of the m-stack. It can be clearly seen that the peak voltages for the first cycles were in the range of 270–450 mV (0.14–0.23 mA) whilst the second cycles were in the range of 260–390 mV (0.13–0.20 mA). Although the peak voltage levels were not fully stabilised between the two cycles, since there is variance with fresh urine, the acclimation period was considered completed as no further increase in peak output levels was recorded.

As can been seen in Fig. 2a, when the cells were arranged in cascades and fed in continuous mode (t = 28.7 h), the voltage levels depicted a sharp increase of 17–27.7%. The enhanced performance remained stable for approximately 22 h whilst the voltage for all MFC units, except m1, subsequently decreased. This result is attributed to the insufficient carbon energy source for cells m2–m12, due to the low flow rate 7.51 ml/h of urine [22].

The data presented in Fig. 2b show the power curves of the cells for the first cycle (t = 5.9 h, Fig. 2a). As can been seen from Fig. 2b (points 1 and 2), a double overshoot effect was observed for all units. This result indicated possible biofilm immaturity on the anode electrodes or sub-optimal ionic conditions in the anodic environment [23]. Overshoot occurs when power and current decrease simultaneously [24]. A similar behaviour was observed during the start–up phase of the t-stack (data not shown).

### 3.2 Voltage/power behaviour of the stacks under different electrical configurations

**Fig. 3:**
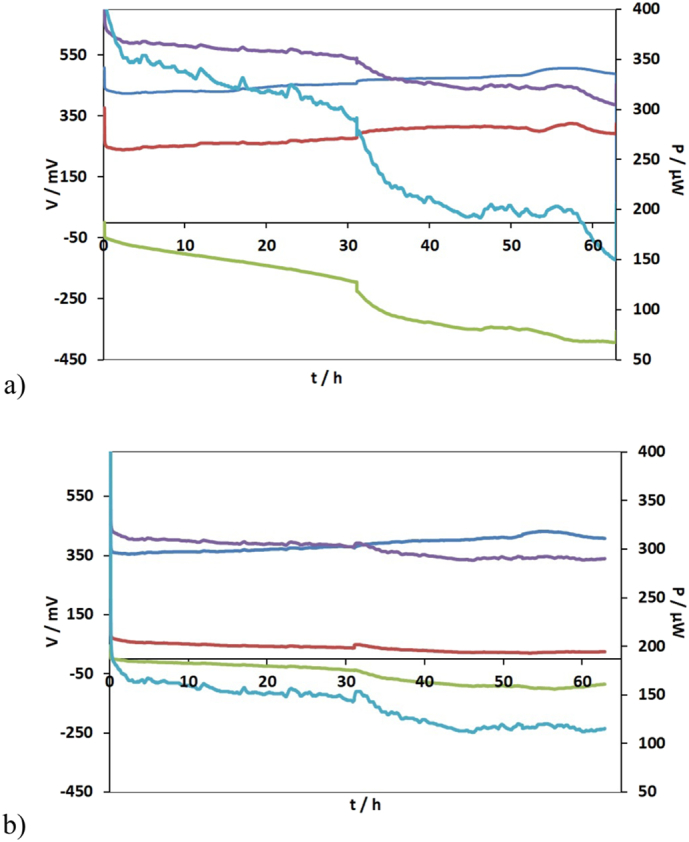
Voltage behaviour of the quadruplets of (a) the m-stack versus time (V/m1–4: , V/m5–8: , V/m9–12: , V/m-stack: , m5: P/m-stack: ) and (b) the t-stack versus time (V/t1–4: , V/t5–8: , V/t9–12: , V/t-stack: , m5: P/t-stack: ). Right axis: Power behaviour of the stacks during time.

**Fig. 4:**
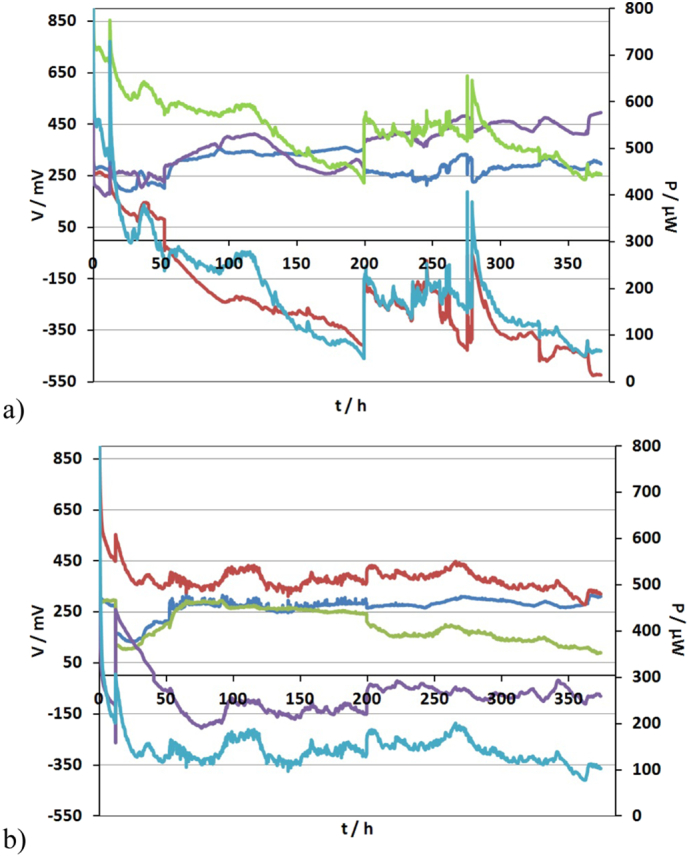
Voltage behaviour of the quadruplets and stack versus time (a) m-stack (V/m1,2,11,12: , V/m3,4,9,10: , V/m5,6,7,8: , V/m-stack: , m5: P/m-stack: ) (b) the t-stack versus time (V/t1,2,11,12: , V/t3,4,9,10: , V/t5,6,7,8: , V/t-stack: , P/t-stack: ). Right axis: Power behaviour of the stacks during time.

The m-stack operated for ~63 h under the first electrical connection whilst the t-stack for almost ~91.1 h. Fig. 3 shows the changes in the monitored voltage V of the quadruples and the stacks and the power output of the m-stack and t-stack, respectively. The voltage of the bottom four MFCs (m9–12; t9–12) in both stacks reversed in polarity and the effect was almost immediate. This result is attributed to substrate imbalance along the MFC cascade. This imbalance caused a disproportional variation in internal resistance among units, which further caused the reversal in polarity [9]. Despite these adverse conditions, the m-stack produced a maximum power of 800 μW whilst the t-stack produced a maximum power of 520 μW after 62.6 h of operation (Fig. 4).

Without exception, all MFCs showed a power overshoot behaviour, which is a sign of biofilm immaturity or suboptimal system performance; given the length of time this experiment has been running for, it was evident that there were intrinsic factors negatively affecting the performance.

**Fig. 5:**
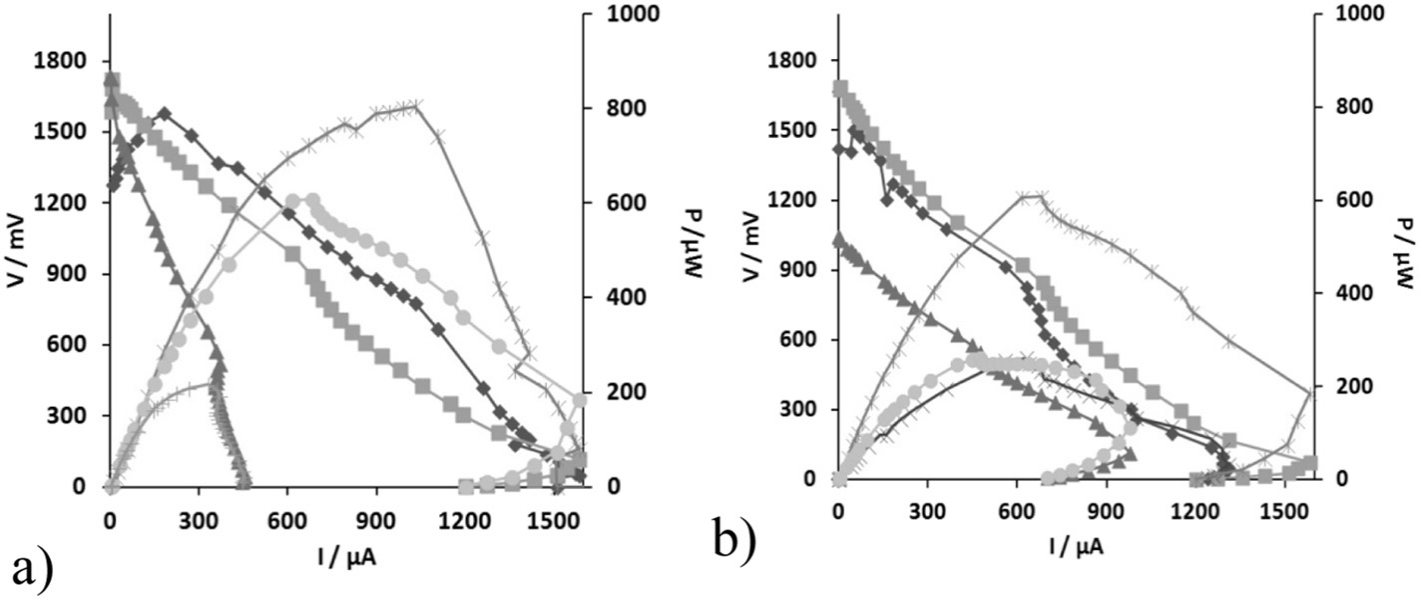
Power and polarisation curves for (a) the m-stack (b) the t-stack. V1 (), V2 (), V3 (), P1 (), P2 () and P3 () represent different points in time during the maturing phase of the stacks.

In order to overcome this barrier, the electrical configuration was changed to compensate for the sequential treatment in the cascade that would inevitably starve the bottom MFCs. Specifically, MFCs 1, 2, 11, 12; 3, 4, 9, 10 and 5, 6, 7, 8, were connected in parallel, respectively. The external load applied to each stack was 1 kΩ. The stacks operated for ~373.9 h under this electrical configuration. Fig. 4 shows the changes in the monitored voltage V of the quadruples and stacks and the power output of the m-stack and t-stack, respectively. Under this electrical configuration the parallel group formed by MFCs 5, 6, 7, 8 reversed in polarity. The maximum power obtained immediately after the new electrical configuration (t = 0 h) was 610 μW for both stacks whereas after 373 h of continuous operation, it decreased to 260 μW and 220 μW for the t-stack and the m-stack, respectively (Fig. 5).

### 3.3 Effect of different electrical connections on the individual MFC units within the stacks

**Fig. 6:**
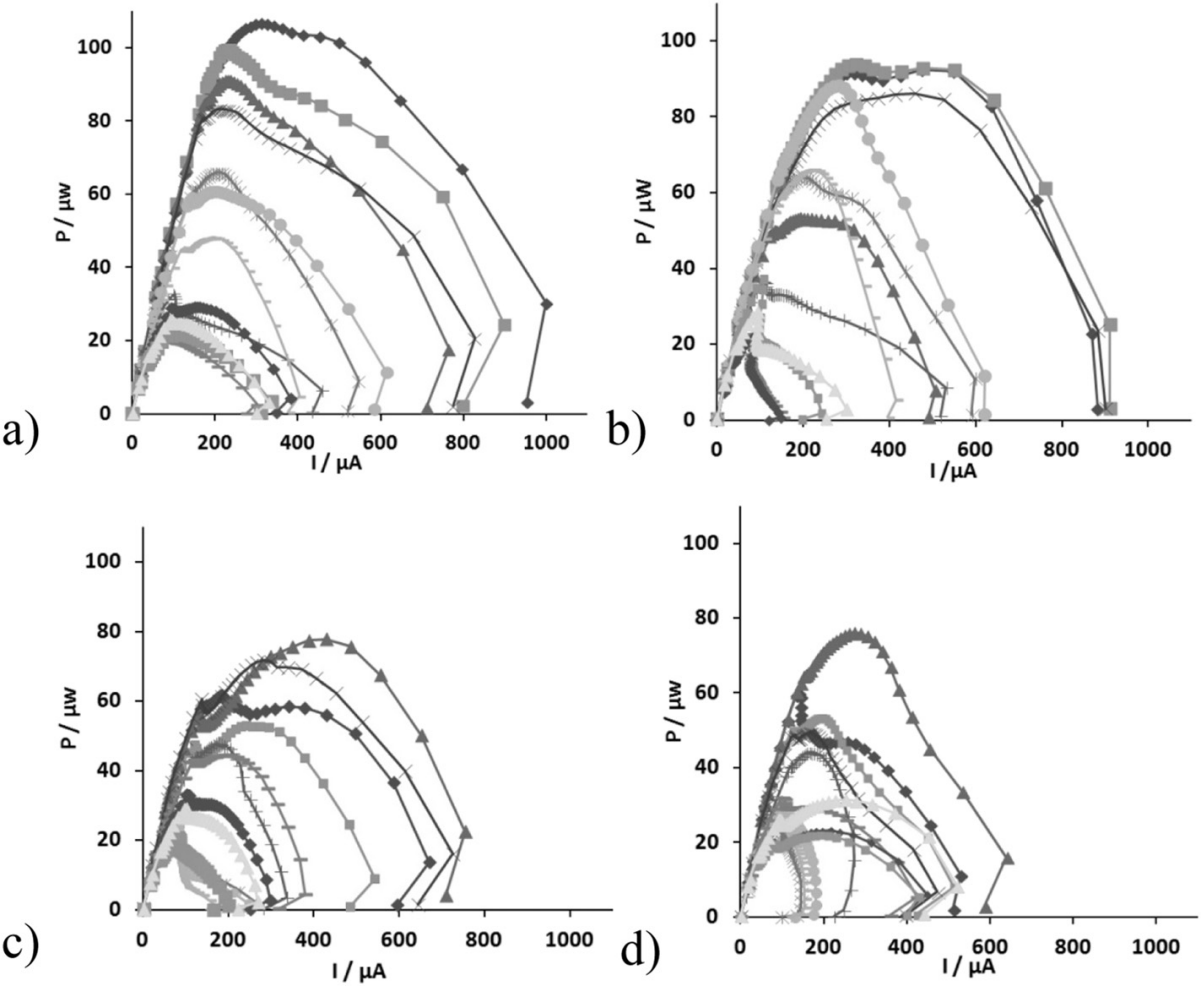
Power curves produced after the MFC units were electrically disconnected from the first connection; the top four (1–4), the middle four (5–8) and the bottom four MFCs (9–12), where connected in parallel, and the three parallel groups were connected in series (3s4p) and the second connection; MFCs 1, 2, 11, 12; 3, 4, 9, 10 and 5, 6, 7, 8, were connected in parallel, and the three parallel groups were then connected in series (a) (b) MFCs from m-stack after the 1st connection and the 2nd connection, respectively (c) (d) MFCs from t-stack after the 1st connection and the 2nd connection, respectively (m1, t1: ; m2, t2: ; m3, t3: ; m4, t4: ; m5, t5: ; m6, t6: ; m7, t7: ; m8, t8: ; m9, t9: ; m10, t10: ; m11, t11: ; m12, t12: ).

In order to determine the effect of different electrical connections on power output of the individual MFC units, two polarisation experiments were conducted at the following times: the 1st was 60 h into the first electrical configuration and the 2nd was again after 60 h under the second electrical configuration, for both stacks. The power curves for the individual MFCs were produced after the cells were electrically disconnected within the cascades (Fig. 6). As can be seen from Fig. 6a, b the power curves generated after the operation of the cells under the two electrical configurations, exhibited a different performance compared to their individual running during the start-up phase (Fig. 2b). In particular, when the mullite cells were electrically connected their performance enhanced whilst there was less of an overshoot phenomenon.

Specifically, during the first electrical connection, the polarisation experiments indicated that the top four (1–4) units produced more power in comparison with the middle four (5–8) and with the bottom four MFCs (9–12) cells. Specifically, the maximum power produced was in the range of: 83–107 μW (maximum power density of 20.8–27 W/m^3^) for the first four cells of the m-stack (m1–m4); 33–66 μW (maximum power density of 8–16.4 W/m^3^) for the middle four (m5–m8); and 24.8–29 μW (maximum power density of 5.4–12.1 W/m^3^) for the bottom four cells (m9–m12) (Table 1). Furthermore, the bottom four MFCs (m9–m12) exhibited a double overshoot phenomenon although the overshoot peaks were less exaggerated in comparison with those generated during the start-up phase (Fig. 6a). The declining performance in the downstream MFCs is attributed to the depletion of organic matter.

**Table 1:** Maximum power produced from mullite cells (m) under two different electrical configurations (MFCs were named based on their position in the cascade i.e. from m1 (the first to receive feedstock) up to m12 (the last to receive feedstock)).

MFC unit:	m1	m2	m3	m4	m5	m6	m7	m8	m9	m10	m11	m12
Pmax (μW)/1st electrical configuration	106.7	99.7	91.2	83.3	65.9	66.7	32.8	48.2	21.8	29.1	21.8	25.8
Pmax (μW)/2nd electrical configuration	92.3	93.8	53.2	86	64	88	36.5	66	22.7	21.9	37.3	28.4

Similar behaviour was observed from the individual MFC units of the t-stack. However, terracotta cells were less powerful compared to the mullite cells when operated under the same conditions. Moreover, a double overshoot phenomenon was observed in almost all cells within the stack (Fig. 6c). In particular, the maximum power produced from MFCs t1–t4, was in the range of 53–78 μW (maximum power density of 13–19.4 W/m^3^), from the middle four, t5–t8, was in the range of 22–48 μW (maximum power density of 5.5–12 W/m^3^) and from the bottom four cells, t9–t12, was in the range of 21–44.5 μW (maximum power density of 5.2–8.2 W/m^3^) (Table 2) The overshoot phenomenon is an indication of suboptimal performance and internal resistance variation, and this is in agreement with previous work [23].

**Table 2:** Maximum power produced from terracotta cells (t) under two different electrical configurations. (MFCs were named based on their position in the cascade i.e. from t1 (the first to receive feedstock) up to t12 (the last to receive feedstock)).

MFC unit:	t1	t2	t3	t4	t5	t6	t7	t8	t9	t10	t11	t12
Pmax (μW)/1st electrical configuration	61.6	53	77.8	71.8	22.5	24	47.7	21.3	44.5	32.8	20.8	29
Pmax (μW)/2nd electrical configuration	59.9	53.4	76.1	49.3	24.7	29.9	44.1	28	32.1	23.1	22	31.4

Similar behaviour of the power curves was observed during the second electrical connection for the individual MFC units of both stacks (Table 1, Table 2) Although the different electrical configurations had an effect on the parallel groups, which reversed in polarity in both stacks, the power curves of the individual MFCs remained on more or less similar values and pattern for both connections (Fig. 6b, d).

### 3.4 Identification of the factors that caused the power decrease

After approximately 453.1 h of continuous operation, the voltage and the power output of both stacks gradually decreased. In order to identify the factors that caused this decrease and to enhance the performance of the stacks, a series of relevant targeted experiments were conducted.

#### 3.4.1 Effect of cathode dehydration

Initially the cathode electrodes of the t-stack were hydrated with deionized water. Following the first hydration of the cathodes, an increase of 37.5% in cell voltage of the t-stack (V_cell_ = 330 mV) was observed. However, this decreased (within 60 h) to its previous values. When the second hydration occurred, a similar increase of 43.5% of cell voltage was observed. The cell voltage dropped again to its previous levels after 60 h of continuous operation, despite a third cathode hydration, 18.3 h after the second hydration. Thus, dehydration of cathodes was possibly one of the contributing factors to the decreasing performance of stacks.

#### 3.4.2 Effect of electrical configuration

Since the level of voltage was not completely recovered to its initial value after the hydration of the cathodes, more tests were performed in order to identify the reasons detrimentally affecting performance. The electrical configuration was shifted to the optimum electrical connection of the cells (all in parallel) and 100 Ω load was applied to each stack. In order to examine if the activity of the biofilm was negatively affected by the voltage reversal, a second enrichment and adaptation of electrochemically active bacteria in the cells was performed in batch mode. During the second inoculation, 50% of anolyte from a separate stack, running under identical conditions, was added mixed with 50% fresh urine, used as feedstock, but no performance improvement was observed.

#### 3.4.3 Effect of catalyst layer coating

The possibility that the activated layer of the cathode electrode coating was degrading (fouling) over time was also examined. In order to assess this, the cathode electrodes were replaced with new identical ones, approximately 2 weeks after the start of the parallel configuration. Once again, no improvement in performance was observed. The replacement of the cathode electrodes was followed by hydration with deionized water, which improved performance by 22%. However, approximately 16 h after this, the voltage returned to previous levels achieved before replacing the cathodes.

#### 3.4.4 Effect of struvite deposition

As already mentioned, the greatest challenge was fuel availability, which resulted in having to run the MFCs at very low – and quite possibly suboptimal – flow rates (7.51 ml/h). This was certainly a contributing factor both to the detriment of performance, but also to the deposition of struvite inside the anode chamber (see Fig. 1d). In order to examine this, and check whether struvite was blocking the biofilm and preventing fuel from percolating through, the cells were opened and the struvite, which accumulated on the anode electrodes was removed, after approximately 1 month (756.1 h) from the start of the parallel configuration; once again there was no significant improvement recorded following this step.

#### 3.4.5 Investigation of ceramic material blockage

The final step of the investigation was to examine if the ceramic material was gradually blocked during time. In order to examine this parameter, the anode and cathode electrodes were replaced by fresh identical ones, whilst keeping the same ceramic materials as the membrane. The cells were inoculated and matured exactly in the same way as before, i.e. in batch mode, under a fixed external load of 2 kΩ for each cell. During inoculation, 50% of activated sewage sludge and 50% of fresh urine was used as feed. These attempts proved unsuccessful, indicating the distinct possibility that the ceramic material itself was blocked by the slow rate of ion transfer and accumulation of struvite. This was probably due to the sub-optimally low flow rate, which would have promoted slow growing organisms and - as proven - a significant accumulation of struvite. It is also possible that the open porosity might have played a key role to the detriment of performance, since it was higher than the open porosity known to work better with ceramic MFCs. The higher porosity may, on the one hand, have facilitated a freer movement of ions and macromolecules, but on the other hand, due to the low rate of electron (and concomitant cation) transfer, the presence of such macromolecules might have resulted in holding-up slowly moving ions, and thus blocking the passage. This may not have been the case if the material porosity was different or the material itself was of a different composition.

Although the performance did not improve, following the various attempts of recovery, it is worth noting that through these experiments (final step in particular) it was shown that the ceramic material can actually block, (just like any other filter/porous material). This is usually if the movement of particles through the material is not continuous or is occurring at an unfavourable rate of transfer. The MFC operation is based on charged movement of electrons and ions, which if continuous and at optimum rates, then the movement across the membrane will also be continuous with minimum or zero ‘stuck’ molecules. Further work will investigate more thoroughly this parameter and study how power output can directly affect (or not) the blockage of the ceramic separator. In addition, the study of factors contributing to the stable performance of the stacks and improving system performance will also be investigated. In particular, the role of ceramic material properties (porosity, composition, geometry and size) on stack performance, in addition to different operating conditions (electrical connection, flow rate) are critical parameters that should be considered for stack improvement and use in practical applications.

## 4 Conclusions

A newly designed, ceramic MFC unit was operated as part of two twelve cell cascade stacks using urine as the feedstock. The best performance occurred during the first electrical connection which produced a maximum power of 0.8 mW after 62.6 h of continuous operation. However, the voltage and the power output of both the m-stack and t- stack gradually decreased. In an attempt to improve performance, a series of targeted tests were performed. These experiments indicated that during continuous operation, mullite and terracotta were blocked by the struvite precipitation, which was the result of flow rate and electrode conformation.
